# Clarity and Emotional Regulation as Protective Factors for Adolescent Well-Being: A Moderated Mediation Model Involving Depression

**DOI:** 10.3390/ejihpe15070130

**Published:** 2025-07-11

**Authors:** Jonathan Martínez-Líbano, María-Mercedes Yeomans-Cabrera, Axel Koch, Roberto Iturra Lara, Patrícia Torrijos Fincias

**Affiliations:** 1 Facultad de Educación y Ciencias Sociales, Universidad Andres Bello, Concepción 4030000, Chile; 2 Escuela de Psicología, Facultad de Salud y Ciencias Sociales, Universidad de Las Américas, Santiago 2531098, Chile; 3 Facultad de Ciencias para el Cuidado de la Salud, Universidad San Sebastián, Concepción 4080871, Chile; 4 Faculty of Education, University of Salamanca, 37008 Salamanca, Spain

**Keywords:** emotional regulation, emotional clarity, adolescent well-being, depression, moderated mediation model

## Abstract

Introduction: Adolescent well-being is influenced by emotional regulation and clarity, particularly in contexts of depression, stress, and anxiety. Objective: This study explores how depression mediates the relationship between emotional regulation and well-being and whether emotional clarity moderates this interaction, providing a comprehensive model to understand adolescent mental health. Methodology: A cross-sectional study was conducted with 636 Chilean adolescents aged 10–18. Emotional clarity and regulation were assessed using the TMMS-24 scale, depression with the DASS-21 scale, and subjective well-being with the Personal Well-Being Index (PWI). Statistical analyses included descriptive statistics, Pearson correlations, and moderated mediation models (PROCESS Macro, Models 4 and 7). Results: Emotional regulation positively correlated with subjective well-being (r = 0.373, *p* < 0.01) and negatively with depression (r = −0.251, *p* < 0.01). Depression partially mediated the relationship between emotional regulation and well-being (B = 0.149, 95% CI [0.082, 0.225]), with regulation explaining 86.41% of the effect. Emotional clarity moderated the regulation-depression link, with higher clarity amplifying the protective impact of regulation (index = 0.008, 95% CI [0.0017, 0.0149]). Conclusions: Emotional regulation and clarity are vital for adolescent well-being and enhance the protective role of regulation against depression. Interventions targeting both constructs could improve mental health outcomes in vulnerable populations.

## 1. Introduction

Currently, it has been evidenced that a large part of the world’s population presents a decrease in the quality of life because of negative alterations in mental health (Cianconi et al., 2023). Specifically, to consider the quality of life construct, variables such as (i) anxiety, a psychological and physiological process resulting from the combination of tension, apprehension, nervousness, and unpleasant thoughts of worry (Spielberger, 2021), (ii) stress, considered a psychological condition that occurs when the person perceives a substantial imbalance between the demands they endure and their ability to meet them (Kent, 2003), and (iii) depression, established as a persistent mood state of excessive sadness and/or a significantly reduced experience of pleasure (Kendler, 2016) which significantly affect the well-being of different groups of the poulation (G. D. Barahona-Fuentes et al., 2019), must be considered. In this regard, special mention should be made of the school population (Martínez-Líbano & Yeomans-Cabrera, 2024). Thus, in this context, it has been shown that alterations in mental health due to anxiety, stress, or depression are associated with drug use and consumption, increased suicidal ideation, low levels associated with physical conditions (G. Barahona-Fuentes et al., 2023), and low academic performance (McCurdy et al., 2023). Recent national and regional studies have reported that more than 50% of Chilean teenagers present depressive symptoms, anxiety, and stress, particularly in the context following the COVID-19 pandemic (Martínez-Líbano et al., 2023; Martínez-Líbano & Yeomans-Cabrera, 2024). Similarly, in Latin America, mental health challenges in the youth population are intensified by structural inequality, violence, and insufficient access to specialized services (Gómez-Restrepo et al., 2025). These epidemiological indicators underscore the urgency of developing and studying protective emotional competencies such as emotional regulation and clarity, which could mitigate the psychological vulnerability observed in this population. Fortunately, different socio-educational strategies have been proposed (Peter & Jule, 2023).

The search for effective strategies to address mental health problems has gained relevance in the scientific field. However, it has not always been given the importance it deserves in education (Tao et al., 2023). Among these strategies, emotional regulation— understood as the process by which individuals manage the intensity and duration of their emotions (Kozubal et al., 2023)—has been widely studied for its impact on preventing emotional disorders and promoting overall well-being by enabling individuals to adaptively manage their emotions and minimize the effects of stressors in their daily lives (Eisenstadt et al., 2021; Morrish et al., 2018). Likewise, other adaptive strategies, such as cognitive restructuring, are associated with positive psychological outcomes, as opposed to less effective strategies, such as emotional suppression, which is linked to an increased risk of mental health problems (Aldao et al., 2010; Carl et al., 2013; Dryman & Heimberg, 2018). However, the specific mechanisms underlying this relationship are still not entirely clear, especially in the context of interactions with other variables, such as emotional clarity and depressive symptoms, which constitute factors that could influence the effectiveness of emotional regulation strategies.

In this sense, emotional clarity—the ability to identify and understand one’s emotions (Boden et al., 2013; Eckland & Thompson, 2023)—is a key component of emotional intelligence that facilitates adaptive emotion regulation. Previous studies (Extremera & Fernández-Berrocal, 2006) have shown that high emotional clarity is associated with lower levels of anxiety and depression, as well as with greater general well-being. However, other works, such as those of Flynn and Rudolph (2010, 2014), have also pointed out that low emotional clarity could increase vulnerability to stressful events, hindering the use of effective emotional regulation strategies and contributing to the development of depressive symptoms. These relationships highlight the significance of examining the influence of emotional clarity on emotional regulation and overall well-being.

Despite emotional regulation being a key predictor of well-being, recent studies suggest that depression may mediate this connection, exacerbating the adverse effects of ineffective emotional regulation and reducing well-being levels in individuals affected by it (Cejudo et al., 2020; Gómez-Romero et al., 2018; Guerra-Bustamante et al., 2019). However, it has not yet been fully explored how depression acts as a mediator in the relationship between emotional regulation and well-being. Similarly, it has been postulated that emotional clarity could act as a moderator, buffering the impact of depression on well-being by facilitating the use of adaptive emotional regulation strategies (Lischetzke & Eid, 2017; Rubenstein et al., 2015). Unfortunately, these hypotheses require further empirical evidence, especially in adolescent and young populations, who are more susceptible to experiencing high levels of stress, anxiety, and depression.

Indeed, understanding how emotional regulation, depression, and emotional clarity interact is crucial from both a theoretical and practical perspective. From a theoretical standpoint, this study aims to fill gaps in the literature by exploring the underlying mechanisms that explain how these variables influence subjective well-being. In particular, it contributes to the advancement of integrative models of adolescent mental health by combining emotional regulation theory with cognitive-behavioral frameworks, re-emphasizing the conditional nature of emotional processes in the presence of psychological symptomatology. Analyzing the mediation of depression and the moderation of emotional clarity would provide a more robust conceptual framework for understanding the relationship between emotional regulation and well-being (Flynn & Rudolph, 2014). From a practical perspective, the results of this study can inform the design of interventions aimed at strengthening emotional clarity and emotional regulation skills, particularly in more vulnerable populations. For example, identifying emotional clarity as a moderator allows practitioners to tailor interventions to individuals who are unlikely to benefit from traditional emotional regulation strategies alone. Similarly, clarifying the mediating role of depression suggests that preventive programs should simultaneously address affective symptoms to enhance their impact on well-being.

Therefore, the primary objective of the present study is to investigate the interplay between emotional regulation, emotional clarity, and depression in influencing subjective well-being among adolescents. To achieve this, we draw upon two well-established theoretical frameworks in contemporary psychology, which provide the conceptual foundation for the proposed relationships.

According to Gross’s Emotion Regulation Theory (Gross, 1998, 2015), individuals employ cognitive and behavioral strategies—either antecedent-focused or response-focused—to manage emotional experiences. Among adolescents, the ability to regulate emotions adaptively plays a crucial protective role against emotional distress, and it is associated with higher levels of well-being and lower symptoms of depression. Emotional regulation is thus conceptualized in this study as both a direct predictor of subjective well-being and an indirect factor via its effects on depression.

From the cognitive-behavioral perspective (Beck, 1979; McIntosh & Fischer, 2000), it is proposed that maladaptive emotional and cognitive processing increases vulnerability to depressive states. Emotional clarity—defined as the ability to understand and label one’s emotional states—is essential in this context, as it enhances metacognitive processing and facilitates the use of effective regulation strategies. Low emotional clarity can lead to cognitive distortions, rumination, and emotion suppression, which in turn contribute to depressive symptomatology.

Based on these frameworks, the present study proposes a moderated mediation model that integrates these emotional and cognitive variables. Specifically, we hypothesize:

**Hypothesis 1** **(H1).** *Depression will mediate the relationship between emotional regulation and subjective well-being, such that greater emotional regulation will be associated with lower depression and, in turn, higher well-being.*

**Hypothesis 2** **(H2).** *Emotional clarity will moderate the relationship between emotional regulation and depression, such that this relationship will be stronger (i.e., more negative) in adolescents with high levels of emotional clarity.*

This integrative approach aims to expand our understanding of adolescent mental health by identifying the mechanisms through which emotional competencies influence well-being and by proposing moderators that either enhance or attenuate these effects.

This study advances current knowledge in several important ways. First, it employs a moderated mediation model (PROCESS Model 7) to investigate the interplay between emotional regulation, emotional clarity, depression, and subjective well-being in adolescents. This approach has not been previously tested in Latin American youth populations. Second, unlike prior studies that typically examine mediation or moderation in isolation, our model captures the conditional indirect effect of emotional regulation on well-being through depression, depending on levels of emotional clarity. Third, we employ the Johnson-Neyman technique to determine the specific values at which emotional clarity significantly moderates this relationship, offering actionable thresholds for intervention design. Finally, this study provides cross-cultural evidence from Chilean adolescents in a post-pandemic context, thereby addressing a key gap in the global literature on adolescent emotional health. Together, these elements offer a comprehensive and contextually grounded model that both replicates and meaningfully extends prior research.

## 2. Methodology

The following research was conducted under the guidelines and recommendations of the Strengthening the Reporting of Observational Studies in Epidemiology (STROBE) statement (Von Elm et al., 2007).

### 2.1. Study Design

The present study used a quantitative, cross-sectional, convenience sample design. Statistical software (G*Power, v3.1.9.7, Heinrich-Heine-Universität, Düsseldorf, Germany) was used to determine an appropriate and robust sample size.

### 2.2. Participants

The combination of tests used in the software established that the moderated mediation model with four variables requires a minimum sample size of 118 participants to detect moderate effects (f2 = 0.15), with a confidence level of 95% (α = 0.05) and a statistical power of 80% (1 – β = 0.801) to answer the stated hypotheses.

The sample comprised 636 schoolchildren (*n* boys = 359; *n* girls = 277) between 10 and 18 years of age (12.92 ± 1807) from various public, subsidized, and private schools in Chile. Specifically, 43% of the participants came from public schools, 38% from subsidized schools, and 19% from private schools. This information helps clarify the socio-educational context of our sample. Participation in this research depended on obtaining the signed assent of the schoolchildren themselves as well as the informed consent of their parents or guardians. This research adhered to the ethical principles outlined in the Declaration of Helsinki (World Medical Association, 2013), local regulations governing research with minors, and the approval of the Ethics Committee of the Universidad Andrés Bello, as per resolution 031/2022.

The inclusion criteria considered for this study were the following: (i) obtaining the informed consent of both parents or guardians and the assent of the participating schoolchildren, (ii) being enrolled in selected educational institutions, and (iii) being within the age range of 10 to 18 years old. The exclusion criteria were as follows: (i) lack of cooperation to complete the questionnaires in their entirety and (ii) schoolchildren with permanent educational needs mentioned in Decree No. 83 of the Chilean Ministry of Education (Ministry of Education, 2015) such as visual, hearing, intellectual or multiple disabilities, dysphasia or autistic disorder.

### 2.3. Measurements

Emotional clarity and emotional regulation: The TMMS-24 scale (Trait Meta-Mood Scale, 24-item version) assessed adolescents’ perception of their emotional clarity and emotional regulation. This scale, initially developed in English (Salovey et al., 1995), was later adapted for use in Spanish (Fernández-Berrocal et al., 2004). This instrument measures three key dimensions: emotional attention, emotional clarity, and emotional regulation. The items are scored on a 5-point Likert scale, ranging from 1 (strongly disagree) to 5 (strongly agree). Higher scores indicate a higher level in each dimension. The scale has demonstrated adequate psychometric properties in the adolescent population (Martínez-Líbano et al., 2025), with a reported Cronbach’s alpha of 0.931 for the scale and factor loadings of 0.904 and 0.883 for emotional clarity and emotional regulation, respectively, in this study.

Depression: The Spanish version of the DASS-21 scale (Depression Anxiety Stress Scale-21 items) was used to measure the participants’ levels of depression (Lovibond & Lovibond, 1995). This instrument includes three subscales that assess symptoms of depression, anxiety, and stress, with seven items per subscale. Each item is scored on a Likert scale from 0 (“Does not apply at all”) to 3 (“Applies a lot or most of the time”). The scale has demonstrated high reliability in the adolescent population, with an overall Cronbach’s alpha of 0.949 and for depression of 0.905. It also features cultural and idiomatic adaptation, demonstrating high reliability and validity among Chilean adolescents (Antúnez & Vinet, 2012).

Subjective well-being was assessed using the Personal Well-Being Index (PWI) (Oyanedel et al., 2015), in its original Spanish version. The Well-Being Scale is widely used to assess satisfaction with life across various domains. This scale comprises seven items that cover key dimensions of well-being, including health, personal relationships, and personal achievement, rated on a Likert scale from 0 (totally dissatisfied) to 10 (totally satisfied). In this study, Cronbach’s alpha for the PWI was 0.955, supporting its reliability in the adolescent population.

### 2.4. Procedure

The researchers visited the selected schools for a week to invite the principals to participate. The following week, with the principals’ approval, they delivered and collected ethical documents for parents or guardians and adolescents who wished to participate in the study. Finally, during the third, fourth, and fifth weeks, the study questionnaires were applied in classrooms in the presence of the researchers. This made it possible to help resolve any potential questions that might arise among the participants. Each data collection session included between 15 and 25 students and lasted approximately 30 min. Participants did not receive financial compensation or academic incentives; their participation was entirely voluntary, and they were thanked for their contribution at the end of each session.

### 2.5. Statistical Analysis

The data were analyzed using MAC SPSS 29.0 software (IBM Corp., 2025) and the PROCESS macro by Hayes (Hayes, 2017) to evaluate mediation and moderated mediation models, which allow for the estimation of direct, indirect, and conditional effects within a single model. The descriptive statistics calculated means, standard deviation (SD) for continuous variables, and frequencies (percentages) for categorical variables. These statistics describe the characteristics of the participants and the main study variables. In addition, to minimize possible biases in the analyses, the following covariates were included: age, coded into age ranges (10–12 = 1, 13–15 = 2, 16–18 = 3), and gender, coded as male (1) and female (2).

Independent t-tests and analysis of variance (ANOVA) were used when appropriate to examine significant differences between groups, controlling for covariates (age and gender). This analysis enabled us to identify potential differences in levels of emotional clarity, emotional regulation, depression, and subjective well-being among demographic groups. Pearson’s correlation analysis was then used to assess the bivariate relationships between emotional clarity, emotional regulation, depression, and subjective well-being. These correlations provided initial insights into the relationships between key variables.

**Figure 1:**
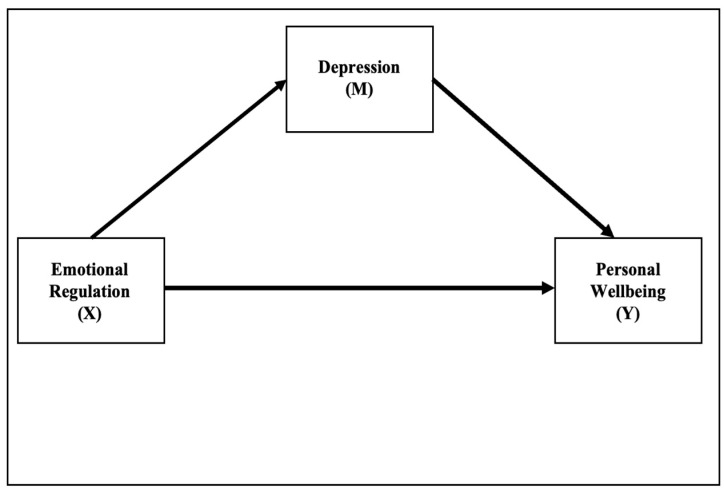
Mediation model between emotional regulation (X), depression (M), and subjective well-being (Y).

Models 4 and 7 of the PROCESS macros for SPSS were applied (Hayes, 2017). Model 4 was used to test the mediating role of depression in the relationship between emotional regulation and subjective well-being. Model 7, a moderated mediation model, was employed to investigate whether emotional clarity moderates the relationship between emotional regulation and depression, thereby influencing the indirect effect on well-being (See Figure 1).

A bias-adjusted 95% confidence interval (CI) was calculated using 5000 bootstrap resamples. If the confidence interval did not include the value 0, the effect was considered significant. A simple slope test was performed to visualize the moderation results, evaluating the conditional effects on the moderator variable’s mean and ± 1 standard deviation (SD) levels (emotional clarity). Additionally, the Johnson–Neyman (J-N) technique was employed to identify the specific values of emotional clarity at which the moderating effect became statistically significant. All estimated effects reported by the PROCESS macro were expressed in terms of standardized regression coefficients (β) to facilitate the interpretation of the results.

## 3. Results

In total, 636 adolescents aged 10 to 18 participated in the study, comprising 277 females (43.6%) and 359 males (56.4%). The mean age of the participants was 12.92 years (SD = 1.807), with participants distributed across the different educational levels of elementary and middle school. The highest proportion of students corresponded to the fifth and seventh grades (20.9% and 20.3%, respectively), while the higher levels, such as the fourth grade, were less represented (0.6%) (see Table 1).

### 3.1. Bivariate Correlations Between Variables

The correlation analysis between emotional regulation, depression, and subjective well-being (see Table 2) showed significant associations that support the proposed theoretical relationships. Concerning emotional regulation and depression, a negative correlation was found (r = −0.251, *p* < 0.01), indicating that higher levels of emotional regulation are associated with lower levels of depressive symptoms. Emotional regulation correlated positively with subjective well-being (r = 0.373, *p* < 0.01), indicating that a better ability to manage emotions is associated with higher life satisfaction. Depression showed a significant negative correlation with subjective well-being (r = −0.337, *p* < 0.01), reflecting that higher levels of depressive symptoms are associated with lower perceived well-being.

### 3.2. Mediation Analyses

Mediation analysis using PROCESS Model 4 (Hayes, 2017) provides evidence that emotional regulation has a significant direct effect on subjective well-being (*p* < 0.001), in addition to a significant indirect effect mediated by depression (B = 0.149, 95% CI: [0.082, 0.225]). These findings highlight the importance of emotional regulation as a key resource for improving subjective well-being, both directly and indirectly, through reducing levels of depression. Mediation was partial, as the direct effect accounted for 86.41% of the total effect, while the mediated effect explained the remaining 13.59%. Details for mediation analyses are presented in Table 3.

### 3.3. Moderated Mediation Analyses

**Figure 2:**
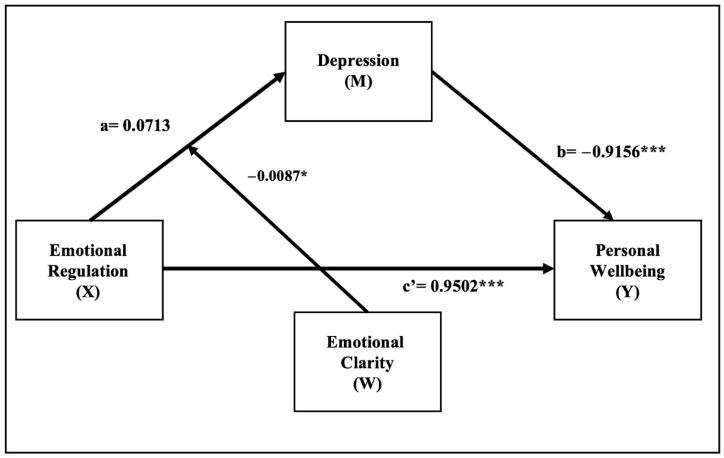
Model of moderated mediation between emotional regulation and personal well-being, moderated by emotional clarity. Note. * *p* < 0.05; *** *p* < 0.001.

Moderated mediation analysis using PROCESS Model 7 revealed that emotional clarity has a significant moderating effect on the relationship between emotional regulation and depression (*p* = 0.0048). Furthermore, the indirect effects of emotional regulation on subjective well-being, mediated by depression, varied by the level of emotional clarity. These findings suggest that, in the young and adolescent population, with greater emotional clarity, the ability to regulate emotions impacts more profoundly on the reduction in depression and, therefore, significantly improves their subjective well-being. Moderate mediation was confirmed by the moderate mediation index (index = 0.0080, 95% CI: [0.0017, 0.0149]). Details for moderated mediation analyses are presented in Table 4 and Figure 2.

### 3.4. Simple Slope Test and J-N Technique Analysis of the Moderating Effect of Emotional Clarity

To further explore the moderating role of emotional clarity in the relationship between emotional regulation and depression, this study categorized emotional clarity into high and low levels, defined as +1 standard deviation (SD) above the mean and −1 SD below the mean, respectively. As shown in Table 5, regardless of the level of emotional clarity, the relationship between emotional regulation and depression was significantly moderated at medium and high levels of emotional clarity (*p* < 0.05). The results of the simple dependent test revealed that as emotional clarity increases, the adverse effect of emotional regulation on depression becomes stronger.

In adolescents with high emotional clarity (+1 SD), the relationship between emotional regulation and depression was stronger (B = −0.2166, 95% CI: [−0.3164, −0.1167], *p* < 0.001). At medium levels of emotional clarity, the relationship was also significant (B = −0.1293, 95% CI: [−0.2030, −0.0556], *p* < 0.001). At low levels of emotional clarity (−1DE), the effect was not significant (B = −0.0595, 95% CI: [−0.1437, 0.0246], *p* = 0.1653).

**Figure 3:**
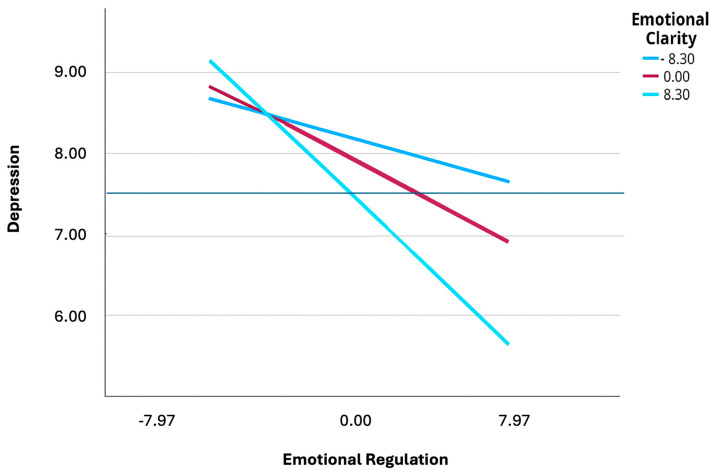
Moderation of emotional clarity in the relationship between emotional regulation and depression.

The findings also indicated a significant cutoff point within the observed range of emotional clarity identified using the Johnson–Neyman technique (Figure 3). The technique showed that the moderating effect of emotional clarity on the relationship between emotional regulation and depression becomes statistically significant when emotional clarity values exceed 17.1781 (approximately the 26th percentile).

## 4. Discussion

### 4.1. Total Effects of Emotional Regulation on Subjective Well-Being

In line with Hypothesis 1, the results confirm that emotional regulation has a positive predictive relationship with subjective well-being in adolescents. This finding highlights the crucial role of emotional regulation as a protective factor contributing to higher life satisfaction and overall positive perception of well-being (Cejudo et al., 2020; Extremera & Rey, 2015; Fonseca-Pedrero et al., 2020), suggesting that the ability to appropriately manage emotions in challenging contexts, such as those experienced by adolescents in the developmental stage, fosters a more positive outlook and adaptive coping strategies (Modecki et al., 2017). This study reinforces that adolescents and young adults can achieve greater psychological well-being by reducing negative emotions associated with stress and promoting more balanced emotional states (Morrish et al., 2018). This aspect takes on special relevance given that recent publications refer to the fact that the mental health of post-pandemic Chilean children and adolescents is increasingly deteriorating (Martínez-Líbano et al., 2023; Martínez-Líbano & Yeomans-Cabrera, 2024).

From a theoretical perspective, these findings align with Gross’s emotional regulation model, which posits that the ability to manage emotions early in the emotional process (e.g., through cognitive reappraisal) is associated with higher levels of psychological adjustment and well-being (Gross, 2015). This conceptual framework emphasizes that adaptive strategies enable the effective modulation of emotional responses, thereby reducing prolonged exposure to stress.

From a clinical perspective, several studies have demonstrated that mindfulness-based programs, such as Mindful Self-Compassion training and Mindfulness-Based Stress Reduction (MBSR), are effective in enhancing emotional regulation and, consequently, reducing symptoms of anxiety, depression, and stress while increasing psychological well-being (Crego et al., 2025). These interventions have demonstrated sustained positive effects even after one year of continuous practice, underscoring their utility as preventive and therapeutic tools in educational and mental health settings.

### 4.2. Mediation of Depression in the Relationship Between Emotional Regulation and Subjective Well-Being

The present study confirmed the partial mediating role of depression in the relationship between emotional regulation and subjective well-being, thus supporting the stated hypothesis. The results demonstrated that emotional regulation impacts subjective well-being both directly and indirectly by reducing levels of depression in adolescents (B = −0.162, *p* < 0.001; B = 0.149, 95% CI: [0.082, 0.225]). In other words, higher levels of emotional regulation are associated with lower depressive symptoms, which in turn translate into higher subjective well-being (Kraaij & Garnefski, 2015). This is consistent with previous research documenting a negative association between emotional regulation and depression (Aldao et al., 2010; Everaert & Joormann, 2020; D. Y. Liu & Thompson, 2017) and a positive association between emotional regulation and well-being (Džida et al., 2023; Fonseca-Pedrero et al., 2020; Gohm & Clore, 2002). In addition, previous studies (Gómez-Romero et al., 2018; Sawyer & Patton, 2018) have noted that depression exerts a direct adverse effect on subjective well-being.

From a theoretical point of view, these findings reinforce psychological models that highlight the interconnection between emotional regulation, depression, and well-being. For example, the Stress Vulnerability Model (Compas & Wagner, 2017; Zubin & Spring, 1977) suggests that emotional regulation can serve as a protective resource, decreasing vulnerability to stress and reducing depressive symptoms. This is particularly relevant in adolescents, who, by developing emotional regulation skills, are less prone to fall into patterns of rumination or hopelessness that increase the risks of depression and consequently improve their perception of well-being (Giménez-Espert & Prado-Gascó, 2018). Similarly, Gross’s Emotional Processing Theory (Gross, 1998, 1999, 2015) emphasizes that the ability to modulate emotional states affects mental health and the general perception of quality of life.

This finding is also consistent with previous evidence in Latin American contexts, where similar mediation mechanisms have been observed. For example, in a study involving Colombian adolescents, researchers found that greater emotional regulation, combined with proactive coping strategies and prosocial behavior, was significantly associated with higher levels of subjective well-being and life satisfaction (Ripoll-Núñez et al., 2020). Although the study did not assess explicit mediation, the results suggest that emotional regulation could act as a key intermediate mechanism.

Similarly, a Chilean study found that emotional variables in the school context, such as teaching skills and working conditions, mediated the relationship between institutional factors and students’ educational outcomes, highlighting the central role of emotional factors in academic well-being and performance (Villagrán et al., 2018).

From a clinical perspective, these results have important implications. Identifying deficits in emotional regulation in adolescents may be key to preventing the development of depressive symptoms, especially in high-risk populations, such as those with a family history of affective disorders or early adverse experiences (McLaughlin & Lambert, 2017). Furthermore, these findings underscore the importance of interventions that address both emotional regulation and depression, as their combined effect can amplify positive impacts on subjective well-being (Gross & Jazaieri, 2014). For example, adolescents with emotional regulation skills can reappraise adverse situations, reducing their emotional impact and decreasing the risk of developing depressive symptoms. (Willner et al., 2022).

Finally, although the partial mediation of depression was significant, the results also suggest the possible influence of other mechanisms, such as resilience (Azpiazu Izaguirre et al., 2021) and perceived social support (Q. Liu et al., 2021). Resilience, the capacity to adapt and recover in the face of adversity, could be an additional resource that enhances subjective well-being. Likewise, emotional regulation can strengthen interpersonal relationships, thereby increasing the perception of social support and self-efficacy (Mesurado et al., 2018), which further reinforces its protective role. Future research could explore these interactions in greater depth to identify other mediating and moderating factors that amplify the impact of emotional regulation on subjective well-being.

### 4.3. Moderating Effect of Emotional Clarity Between Emotional Regulation and Depression

From the theoretical perspective, after establishing the mediating role of depression, this study revealed a significant moderating effect of emotional clarity on the relationship between emotional regulation and depression, thus supporting Hypothesis 2. Specifically, adolescents and young adults with higher levels of emotional clarity showed a stronger negative relationship between emotional regulation and depression. This finding highlights that individuals who clearly understand and recognize their emotional states are better equipped to benefit from emotional regulation strategies, leading to lower depressive symptoms (Aldao et al., 2010; Mesurado et al., 2018; Park & Naragon-Gainey, 2020).

The moderating role of emotional clarity aligns with emotional regulation theory, which emphasizes that the effectiveness of regulation depends not only on the ability to modulate emotions but also on the ability to understand them (Haas et al., 2019). Emotional clarity enhances people’s ability to process and interpret emotional experiences, thereby amplifying the effectiveness of regulatory strategies (Gratz & Roemer, 2004). This supports the idea that emotional clarity acts as a protective factor, reducing vulnerability to depression when effective emotional regulation strategies are employed (Haas et al., 2019).

From the clinical perspective, these results suggest that adolescents with high emotional clarity benefit more from emotional regulation strategies, as they are better equipped to identify and differentiate their affective states (Cooper et al., 2018). Conversely, adolescents with low emotional clarity may need additional support, as their inability to understand their emotions may limit the effectiveness of their regulatory strategies (Cooper et al., 2018; Gratz & Roemer, 2004). This underscores the need to tailor interventions to individual levels of emotional clarity, ensuring that all adolescents benefit from formative programs designed to enhance their emotional health (Rubenstein et al., 2015).

In conclusion, this study demonstrates that emotional clarity significantly moderates the relationship between emotional regulation and depression. Adolescents with high emotional clarity benefit more from their regulatory strategies, highlighting the importance of fostering both skills to mitigate depressive symptoms effectively. In sum, we can affirm that the potential of these findings provides us with valuable information to develop tailored interventions that address the emotional health needs of adolescents in clinical and educational settings. The results enable us to focus on the interventions, identifying content areas to be addressed and methodological strategies tailored to the beneficiaries.

Therefore, it is necessary to reiterate the importance of implementing comprehensive programs that promote Social Emotional Learning (SEL) in school settings, with a specific focus on strengthening emotional regulation and clarity (Domitrovich et al., 2017). These programs can serve as preventive tools by teaching practical strategies for managing negative emotions and appropriately recognizing and labeling their emotional states to adolescents and young adults. Including emotional literacy components within Social Emotional Learning (SEL) would not only contribute to a significant reduction in the incidence of depressive symptoms but also enhance subjective well-being by equipping individuals with the skills to manage stress and the emotional demands of everyday life. This integrated approach addresses the prevention of emotional problems. It promotes healthy socioemotional development, enhancing the quality of life and psychological well-being of young people during a critical stage in their development. Prioritizing programs that combine strategies for emotional regulation and clarity could be key to maximizing their positive impact, setting a clear path toward stronger mental health in adolescence.

The results of this study confirm the proposed moderated mediation model and offer empirical support for the integration of theoretical constructs derived from Emotion Regulation Theory (Gross, 1998, 2015) and cognitive-behavioral models of depression (Beck, 1979). Specifically, we found that depression partially mediates the relationship between emotional regulation and subjective well-being and that this mediating effect is conditioned by the level of emotional clarity, which acts as a significant moderator.

These findings suggest that adolescents who are better able to regulate their emotions experience lower levels of depressive symptoms, which in turn enhances their overall well-being. However, this effect is significantly more substantial in those who also demonstrate high emotional clarity. This aligns with Gross’s model, which highlights the role of emotional awareness and appraisal as antecedents to regulation strategies, and with Beck’s model, which emphasizes how misinterpretation of emotional cues contributes to affective dysregulation and depressive symptoms.

Methodologically, the use of PROCESS Model 7 and the Johnson–Neyman technique allowed us to identify specific thresholds of emotional clarity at which the protective effect of regulation becomes most effective. This conditional process underscores the importance of targeting both emotional clarity and regulation skills in interventions rather than addressing these competencies in isolation.

Taken together, the results bridge theory and practice by demonstrating that adolescents benefit most from emotional regulation when they also possess the cognitive-emotional skill to understand and label their emotional states. These insights are particularly relevant in post-pandemic educational settings, where interventions need to be both precise and scalable.

### 4.4. Limitations

Among the study’s main limitations is that the cross-sectional design prevents the establishment of causal relationships between the variables investigated. Future longitudinal studies would be necessary to validate the direction of the proposed effects. The sample was selected by convenience, which may limit the generalizability of the findings to other educational and geographic contexts. It is recommended that this study be replicated in more representative and diversified samples. The measures used were based on self-reports, which could have introduced social desirability biases or errors in the participants’ perceptions. Including complementary methods, such as direct observations or data from external informants, could strengthen future studies. The study was conducted exclusively in the Chilean context, which limits the generalizability of the findings to other countries or cultures. Conducting research in diverse cultural contexts to compare the results would be relevant. Although age and gender were included as covariates, the analyses did not include other potentially relevant variables, such as socioeconomic context, quality of family relationships, or academic level. Although the general psychometric properties of the scales used were reported, additional studies may be necessary to confirm their specific validity in Chilean adolescent populations. Possible additional interactions between contextual and emotional variables, such as the role of social support, which could provide a more complete perspective of the factors influencing subjective well-being, were not explored.

Furthermore, future studies could benefit from the inclusion of other validated instruments to assess emotional regulation, such as the Cognitive Emotion Regulation Questionnaire (CERQ), which distinguishes between different cognitive strategies in response to stressful events. This would allow a more detailed analysis of the regulatory mechanisms involved and could enhance the explanatory power of the model.

### 4.5. Future Studies

Although this study provides cross-sectional evidence of the moderating role of emotional clarity, longitudinal studies are necessary to investigate how this relationship evolves over time and whether interventions that enhance clarity can strengthen the protective effects of emotional regulation on depression. Beyond emotional clarity, other factors, such as resilience or social support, may also moderate the relationship between emotional regulation and depression. Future research should investigate these variables to gain a more comprehensive understanding of the underlying mechanisms.

Cultural norms and values about emotional expression and emotional suppression may influence emotional clarity and emotional regulation. Studies in different cultural contexts may show how these processes vary across populations.

## Figures and Tables

**Table 1:** Results descriptive statistics.

Categories	Categories	*n* (%)	Emotional Clarity		Emotional Regulation	
		M ± SD	M ± SD	*t*/F	M ± SD	*t*/F
Gender	Woman	277 (43.6)	22.0 ± 8.64	−4.01 **	24.2 ± 8.33	−3.08 **
	Man	359 (56.4)	24.7 ± 7.84		26.2 ± 7.58	
Age (years)	10	50 (25.4)	25.4 ± 7.00	1.67	25.8 ± 7.01	1.05
	11	120 (24.9)	24.9 ± 7.98		26.5 ± 8.18	
	12	104 (22.2)	22.2 ± 8.18		25.1 ± 7.94	
	13	128 (24.1)	24.1 ± 8.34		25.8 ± 7.90	
	14	100 (22.3)	22.3 ± 8.21		24.7 ± 7.63	
	15	77 (23.2)	23.2 ± 8.81		24.9 ± 8.06	
	16	42 (22.9)	22.9 ± 8.93		23.7 ± 8.99	
	17	13 (21.0)	21.0 ± 8.90		21.8 ± 7.47	
	18	2 (27.5)	27.5 ± 17.6		27.5 ± 17.6	
Grade	Fifth	133 (20.9)	24.6 ± 7.57	0.9	25.2 ± 7.38	1.37
	Sixth	105 (16.5)	23.9 ± 7.79		26.8 ± 7.67	
	Seventh	129 (20.3)	23.6 ± 8.80		26.0 ± 8.30	
	Eight	108 (17.0)	22.6 ± 8.20		24.7 ± 7.99	
	First Medio (Ninth)	70 (11.0)	22.3 ± 7.84		23.7 ± 7.21	
	Second Medio (Tenth)	65 (10.2)	23.9 ± 9.96		25.3 ± 9.23	
	Third Medio (Eleventh)	22 (3.5)	22.5 ± 8.98		24.1 ± 8.84	
	Fourth Medio (Twelfth)	4 (0.6)	21.5 ± 2.88		22.0 ± 5.09	

Note 1. ** *p* < 0.01: Statistical significance levels; M: mean; *n*: number of participants; SD: standard deviation; *t* = independent test for the gender variable. F = one-way ANOVA for the variables age and school year.

**Table 2:** Correlation matrix (*n* = 636).

Variables	Emotional Regulation	Depression	Personal Well-Being
Emotional Regulation	1.000		
Depression	−0.251 **	1.000	
Personal Well-being	0.373 **	−0.337 **	1.000

Note: ** *p* < 0.01.

**Table 3:** Mediation analyses.

Effect	B	SE	*t*	*p*	IC 95% (LCL–UCL)
Total effect (Path c)	1.099	0.107	10.29	<0.001	0.890–1.309
Direct effect (Path c′)	0.950	0.106	8.94	<0.001	0.742–1.159
Indirect effect (Path a × b)	0.149	0.036	—	—	0.082–0.225

Notes: B: unstandardized regression coefficient; IC 95%: 95% confidence interval; LCL: lower confidence limit; *p*: probability value (significance level); SE: standard error; *t*: *t*-value; UCL: upper confidence limit.

**Table 4:** Moderated mediation analyses.

Effect	B	SE	*t*	*p*	IC 95% (LCL–UCL)
Interaction	−0.0087	0.0031	−28.300	0.0048	−0.0148–−0.0027
Conditional (Emotional clarity = 15)	−0.0595	0.0428	−13.891	0.1653	−0.1437–0.0246
Conditional (Emotional clarity = 23)	−0.1293	0.0375	−34.461	0.0006	−0.2030–−0.0556
Conditional (Emotional clarity = 33)	−0.2166	0.0508	−42.601	<0.001	−0.3164–−0.1167
Moderate mediation index	0.0080	0.0034	—	—	0.0017–0.0149

Notes: B: unstandardized regression coefficient; IC 95%: 95% confidence interval; LCL: lower confidence limit; *p*: probability value (significance level); SE: standard error; *t*: *t*-value; UCL: upper confidence limit.

**Table 5:** Effect of emotional regulation on depression at different levels of emotional clarity.

Emotional Clarity	B	SE	*t*	*p*	LLCI	ULCI
Mean − SD	−0.0595	0.0428	−13.891	0.1653	−0.1437	0.0246
Mean	−0.1293	0.0375	−34.461	0.0006	−0.2030	−0.0556
Mean + SD	−0.2166	0.0508	−42.601	0.0000	−0.3164	−0.1167

Notes: B: unstandardized regression coefficient; IC 95%: 95% confidence interval; LCL: lower confidence limit; *p*: probability value (significance level); SE: standard error; *t*: *t*-value; UCL: upper confidence limit.

## Data Availability

The data presented in this study are not publicly available due to privacy and ethical restrictions. However, they may be available from the corresponding author upon reasonable request, provided that appropriate ethical approval has been obtained.
